# P-2025. Understanding the Risk Factors for Pneumonia Readmissions: A Quality Initiative at a Regional Medical Center

**DOI:** 10.1093/ofid/ofaf695.2189

**Published:** 2026-01-11

**Authors:** Jaeden McKenzie, Brigette Anne Suerig, Jaime Jaronko, Nicholas Csikesz, Kamran Manzoor, Frank Schembri, Jessica Viola

**Affiliations:** Tufts University School of Medicine, Boston, MA, Quincy, Massachusetts; Tufts University School of Medicine, Boston, MA, Quincy, Massachusetts; South Shore Health, Boston, Massachusetts; South Shore Health, So. Weymouth, MA, Weymouth, Massachusetts; Tufts University School of Medicine, South Shore Health, Boston, Massachusetts; South Shore Health, Boston, Massachusetts; South Shore Health, So. Weymouth, MA, Weymouth, Massachusetts

## Abstract

**Background:**

Pneumonia accounts for 1 million hospitalizations annually with a 30-day readmission rate of 17%-25%, driven by comorbidities and care coordination challenges. We aim to identify risk factors, compliance issues, and causes of pneumonia readmissions to develop targeted strategies to improve patient care.

**Figure:**
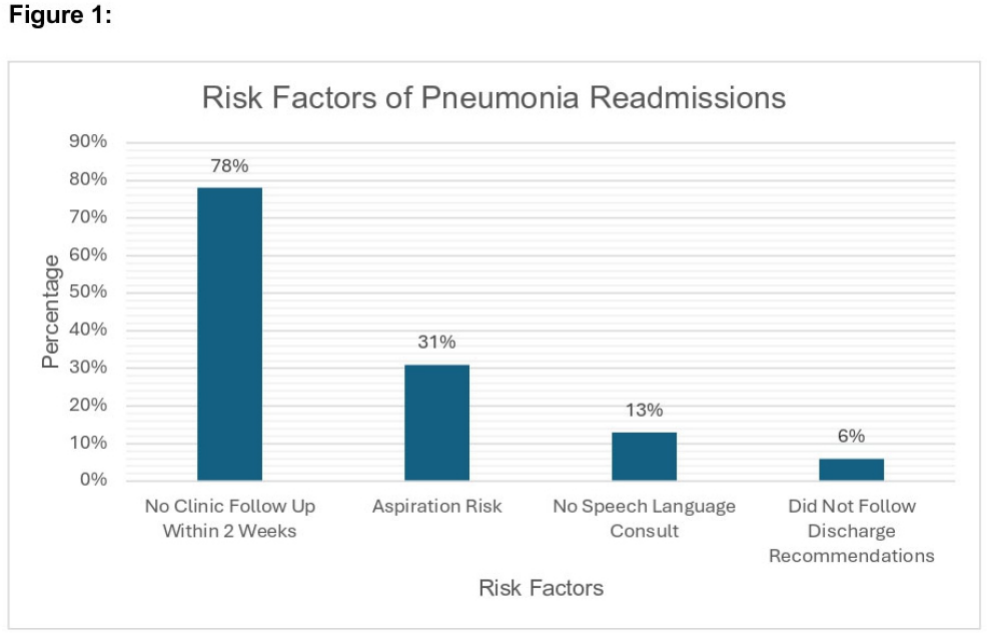

**Methods:**

A retrospective review of electronic health records (EHR) was conducted at a 400-bed regional medical center with pneumonia index admissions over an 11-month period (January-November 2024). Data collected included demographics, comorbidities, clinical presentation, hospital course, discharge planning, and follow-ups. Readmissions within 14 days, rather than standard 30 days, were analysed to focus on deficiencies related to their inpatient care. Analyses was conducted by a multidisciplinary team for optimizing care pathways, antibiotic selection, discharge planning, patient education, and follow-up protocols.

**Results:**

EHR identified 727 cases of hospitalization due to pneumonia in 2024, with 50 patients readmitted within 14 days of discharge. The most frequent comorbidities among readmitted patients were congestive heart failure, coronary artery disease, chronic obstructive pulmonary disease, and chronic kidney disease. Though all patients were treated with appropriate antibiotic coverage, aspiration/dysphagia was identified as a unique aspect of readmissions (15 readmissions [31%]). Additionally, 39 out of 50 patients (78%) did not have a 14-day follow-up with their primary care provider, and 3 out of 50 patients (6%) did not follow discharge recommendations. Readmission was a marker of poor prognosis, with 14 out of 50 patients (28%) deceased at the time of chart review.

**Conclusion:**

Managing patients’ comorbid conditions is pivotal to reducing the burden of pneumonia-related readmissions. This analysis highlights the overall illness burden in patients in patients readmitted with pneumonia. Any intervention that successfully reduces readmissions must address comorbidities, speech language pathology, compliance and coordinated outpatient follow-up including patient education.

**Disclosures:**

All Authors: No reported disclosures

